# Pancreatic and psoas abscesses as a late complication of intravesical administration of bacillus Calmette-Guerin for bladder cancer: a case report and review of the literature

**DOI:** 10.4076/1752-1947-3-7323

**Published:** 2009-09-15

**Authors:** Miguel Álvarez-Múgica, Jesús M Fernández Gómez, Verónica Bulnes Vázquez, Antonio Jalón Monzón, José M Fernández Rodríguez, Laura Rodríguez Robles

**Affiliations:** 1 Department of Urology, Hospital Universitario Central de Asturias, Oviedo, Spain; 2 Department of Radiology, Hospital Universitario Central de Asturias, Oviedo, Spain; 3 Department of Internal Medicine, Hospital Universitario Central de Asturias, Oviedo, Spain

## Abstract

**Introduction:**

Bacillus Calmette-Guerin (BCG) is a live attenuated strain of *Mycobacterium bovis* that has been used to treat urothelial carcinoma since 1976, and has been reported to eradicate disease in more than 70% of patients with *in situ* and stage I disease. To the best of our knowledge, we report the first case of disseminated bacillus Calmette-Guerin infection causing multiple abscesses affecting the pancreatic head and right psoas muscle, diagnosed 5 years after intravesical treatment with bacillus Calmette-Guerin therapy for bladder cancer.

**Case presentation:**

An 83-year-old Caucasian man was hospitalized with a 2-month history of back pain, anorexia, generalized weakness and a 47-pound weight loss. He had previously undergone two transurethral resections for high-grade transitional cell carcinoma of the bladder and had received 12 intravesical bacillus Calmette-Guerin instillations without any complications. He complained of abdominal pain in his right flank. A computed tomography scan of the abdomen showed multiple abscesses affecting the pancreatic head and right psoas muscle. Growth of *Mycobacterium bovis* was determined in cultures of the purulent material obtained by surgical drainage of the abscesses.

**Conclusions:**

This case illustrates the fact that although intravesical administration of bacillus Calmette-Guerin is generally considered to be safe, it is not exempt from complications and these could appear immediately after treatment or as a delayed complication many years later.

## Introduction

Bacillus Calmette-Guerin (BCG) is a live attenuated strain of *Mycobacerium bovis* that has been used to treat urothelial carcinoma since 1976 and has been reported to eradicate disease in more than 70% of patients with *in situ* and stage I disease [1]. Although intravesical therapy with BCG is generally considered safe, serious complications including hematuria, granulomatous pneumonitis, suppurative lymphadenitis, distant intramuscular and bone abscesses, hepatitis, and life-threatening BCG sepsis have been documented [2]. The reported incidence of complications other than minor ones is under 5% [3]. To the best of our knowledge, we report the first case of disseminated BCG infection causing multiple abscesses affecting the pancreatic head and right psoas muscle diagnosed 5 years after intravesical treatment with BCG therapy for bladder cancer.

## Case presentation

An 83-year-old Caucasian man was hospitalized with a 2-month history of back pain, anorexia, generalized weakness and a 47-pound weight loss. The patient had previously undergone two transurethral resections for high-grade urothelial carcinoma of the bladder, receiving 12 intravesical BCG instillations without any complications. Due to tumor progression, he had undergone a radical cystoprostatectomy 5 years earlier and had also had an endovascular stent-graft repair of an infrarenal abdominal aortic aneurysm. The patient complained of abdominal pain in his right flank.

**Figure 1:**
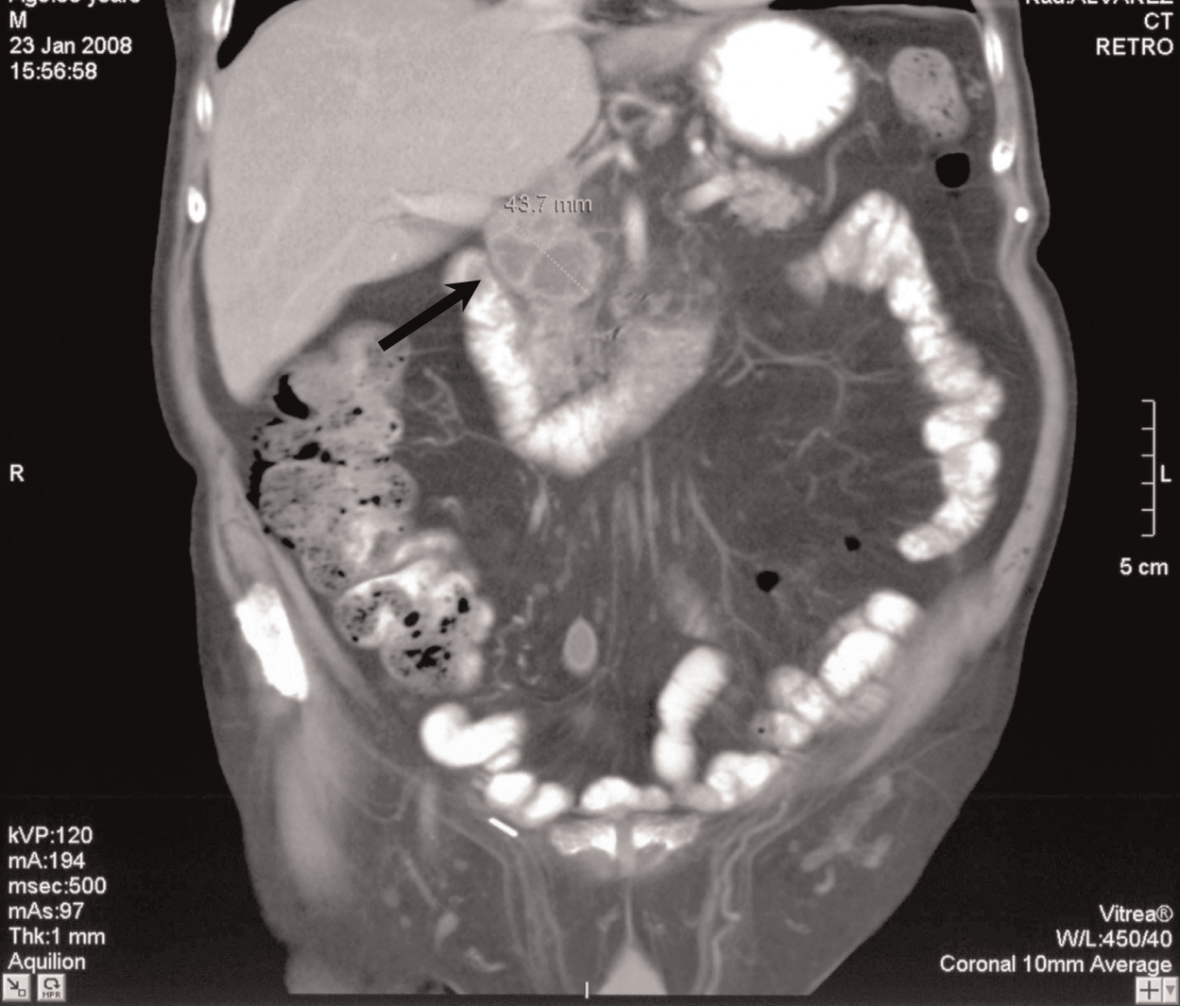
**Computed tomography scan showing 43 mm abscess affecting the pancreatic head**.

**Figure 2:**
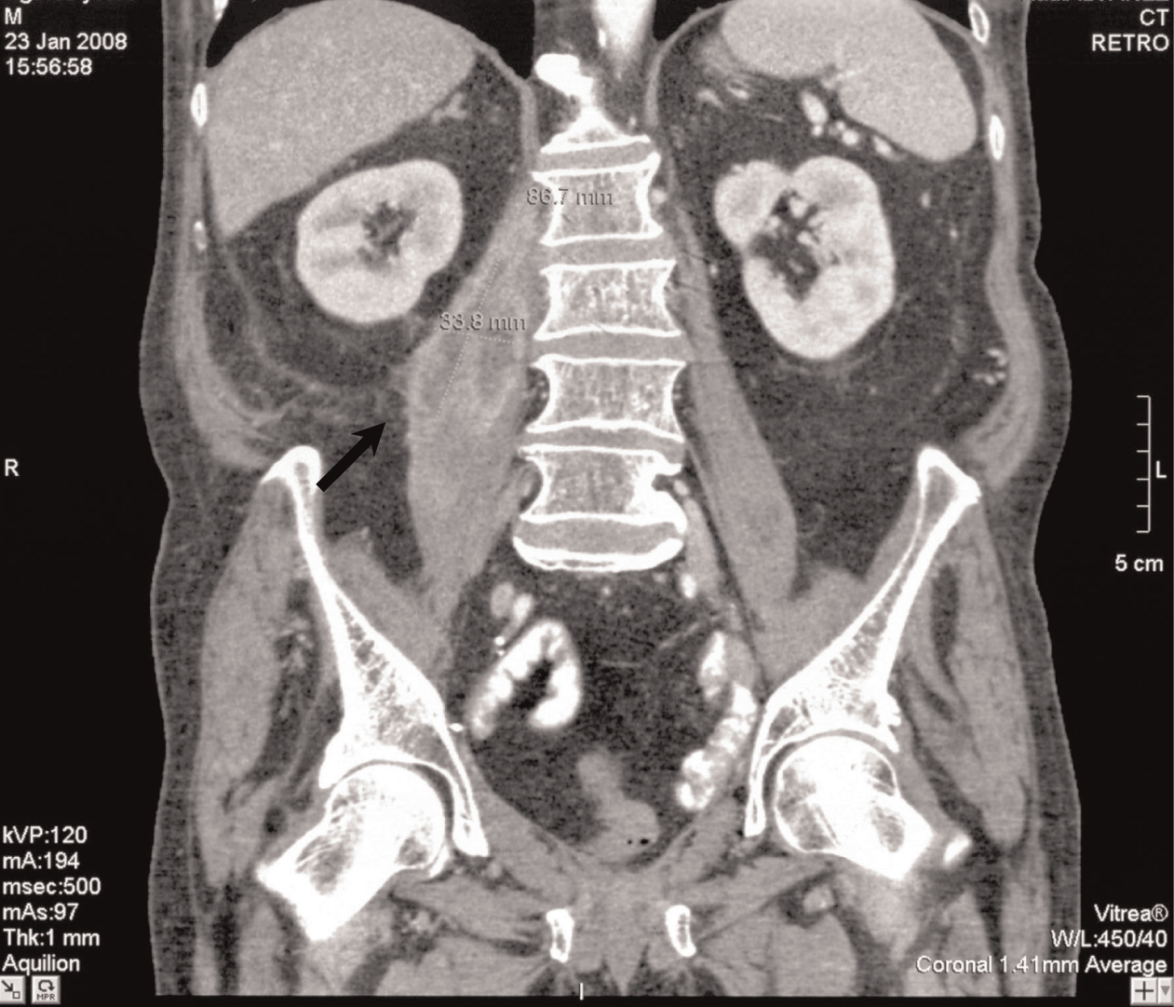
**Computed tomography scan showing right psoas abscess**.

A physical examination revealed general poor health but pulmonary and cardiac examinations were unremarkable and the patient was afebrile. Laboratory investigations were normal with the exception of anemia, a mild renal insufficiency, a left shift of the leucocyte differential and elevated liver enzymes. A computed tomography (CT) scan of the abdomen showed multiple abscesses affecting the pancreatic head (Figure 1) and right psoas muscle (Figure 2). Surgical exploration and drainage were performed and cultures of the purulent material were positive for *M. bovis*. Urine and blood cultures were negative for acid-fast bacilli stains. Antitubercular treatment with isoniazid 300 mg/day, rifampicin 600 mg/day and ethambutol 1200 mg/day was administered for 2 months maintaining isoniazid and rifampicin for a further 4 months. Clinical and laboratory findings improved and after 3 weeks, laboratory tests were normal. A follow-up abdominal CT scan obtained 8 weeks after starting the treatment showed a marked improvement in the extension of the psoas abscess and the number of masses present. The patient remains clinically well after 6 months of the antituberculous therapy.

## Discussion

Intravesical administration of BCG has proved to be an effective form of treatment for some stages of bladder cancer [4]. There are specific risks usually because the virulence is attenuated but the bacillus is still viable and possesses allergic properties. Infrequent, serious complications of this treatment have become apparent as its use has become more widespread. Local complications of BCG therapy for bladder cancer include cystitis, prostatitis, epididymo-orchitis, granulomatous lymphadenitis, ureteral obstruction [5], and more rarely, prostatic abscess [6] and infection of the glans penis [7]. Systemic side effects include fever, influenza-like symptoms, malaise and chills, pneumonitis, hepatitis, rash, arthralgia and arthritis, renal abscess, cytopenia and sepsis. The frequency of these adverse effects was reported by Lamm *et al.*[2] in a study of more than 1200 patients who received this type of immunotherapy. The results revealed only a 2.9% incidence of high fever (>39°C), 1.0% major hematuria, 0.9% granulomatous prostatitis, 0.7% granulomatous pneumonitis and/or hepatitis, 0.5% arthritis or arthralgia, 0.4% epididymo-orchitis, 0.4% life-threatening BCG sepsis, 0.3% urethral obstruction, 0.2% bladder contracture, 0.1% renal abscess and 0.1% cytopenia.

There are also rare cases described in the literature of some other major complications such as mycotic abdominal aortic aneurysm [8], infection of an implantable defibrillator [9], vertebral osteomyelitis [10] and bilateral panuveitis [11]. No infection of the pancreatic head was found in a review of the literature on PubMed.

The mechanism by which BCG exerts its antitumor activity is unknown, but it has been suggested that a non-specific immune response to BCG might also destroy tumor cells [2]. Another suggested mechanism is that the severe inflammation caused by BCG leads to local ischemia, thereby killing tumor cells [3]. Hematogenous spread of BCG and immunoallergic reactions are the two main mechanisms behind the development of systemic complications [2]. Hematogenous spread through an inflamed and/or disrupted urothelium is most frequently caused by traumatic catheterization, bladder perforation, or by extensive tumor resection [3]. In acutely ill patients, triple antituberculous therapy is recommended for 6 months [2]. The use of corticosteroids has risks but the demonstrated absence of organisms in many patients with diffuse granulomas suggests that these complications may be the result of type IV hypersensitivity reactions [12]-[14].

## Conclusions

Although major complications from intravesical BCG treatment are rare, it may result in prolonged fever, pneumonitis, arthritis, vertebral osteomyelitis, hepatitis or life-threatening sepsis. Symptomatology is probably produced jointly by dissemination of *M. bovis* to the reticuloendothelial system and a hypersensitivity response. Antituberculosis agents other than pyrazinamide in combination with corticosteroids are the treatment of choice for disseminated BCG infection. A CT scan is the diagnostic tool of choice in cases of abscesses, with rapid evaluation followed by adequate drainage and antituberculosis agents as the key to the survival of the patient. Our patient improved clinically and although he had recovered fully after 2 months of treatment, he completed the scheduled 9-month treatment. This case reminds us that although intravesical administration of BCG has proved to be a generally safe treatment, it is not exempt from complications and these could appear immediately after treatment or as a delayed complication many years later.

## Abbreviations

BCG: bacillus Calmette-Guerin; CT: computed tomography.

## Consent

Written informed consent was obtained from the patient for publication of this case report and any accompanying images. A copy of the written consent is available for review by the Editor-in-Chief of this journal.

## Competing interests

The authors declare that they have no competing interests.

## Authors' contributions

MAM analyzed and interpreted the patient's medical history and drafted the manuscript. JF made a detailed review of the literature. VB reviewed and interpreted the images from the CT scan. AJ contributed to the overall review and the English translation of the text, and finally JM and LR made the overall review and corrections required. All authors read and approved the final manuscript.
